# Editorial: The impact of social isolation and loneliness on mental health and wellbeing

**DOI:** 10.3389/fpubh.2022.1106216

**Published:** 2022-12-14

**Authors:** Hiroshi Kadotani, Isa Okajima, Keming Yang, Michelle H. Lim

**Affiliations:** ^1^Department of Psychiatry, Shiga University of Medical Science, Otsu, Japan; ^2^Behavioral Sleep Medicine and Sciences Laboratory, Department of Psychological Counseling, Faculty of Humanities, Tokyo Kasei University, Tokyo, Japan; ^3^Department of Sociology, Durham University, Durham, United Kingdom; ^4^Iverson Health Innovation Research Institute, Swinburne University of Technology, Hawthorn, VIC, Australia; ^5^Prevention Research Collaboration, Faculty of Medicine and Health, Sydney School of Public Health, University of Sydney, Sydney, NSW, Australia

**Keywords:** social isolation (SI), loneliness, mental health, wellbeing, COVID-19

Loneliness and social isolation are critical for health and wellbeing. Social isolation is a well-established social determinant of health, and its ill effects have been well-recognized for decades. Over the last 20 years, researchers have increasingly advocated that our health and wellbeing are not only detrimentally affected by being alone but also by feeling lonely (i.e., subjective social isolation) (1). Loneliness was flagged as a critical issue after the onset of the current public health crisis and was recently found to be a prevalent issue across the world (2). Although loneliness is studied as a phenomenon across different nations and cultures, and within different social groups, the exact meaning of loneliness, its antecedents, and its consequences on mental health and wellbeing may vary (3).

The way in which loneliness and social isolation contribute to mental health and wellbeing may be different during the COVID-19 pandemic. This was particularly evident after public health measures such as social restrictions, including national or localized lockdowns, were implemented. Furthermore, quarantine or self-isolation was also recommended for reducing infection (4). It is plausible that many people may have experienced the distress associated with social isolation or loneliness, or both, for the very first time during periods of lockdown, quarantine, and self-isolation. The impacts of quarantine or self-isolation may vary by population. In some populations, self-isolation due to COVID-19 had little influence on daytime sleepiness, insomnia, or depression compared with 1 year earlier (5).

Research interest in this topic has accelerated, with the number of publications about “social isolation” or “loneliness” jumping significantly since 2020 (Figure 1). This reflects the public and research community interest in loneliness and social isolation during the COVID-19 pandemic. The estimated number of publications about “social isolation” or “loneliness” in 2022 decreased from that of 2021 (Figure 1). This decrease may reflect lower interest due to the lower incidence of new COVID-19 cases since January 2022 (6).

**Figure 1:**
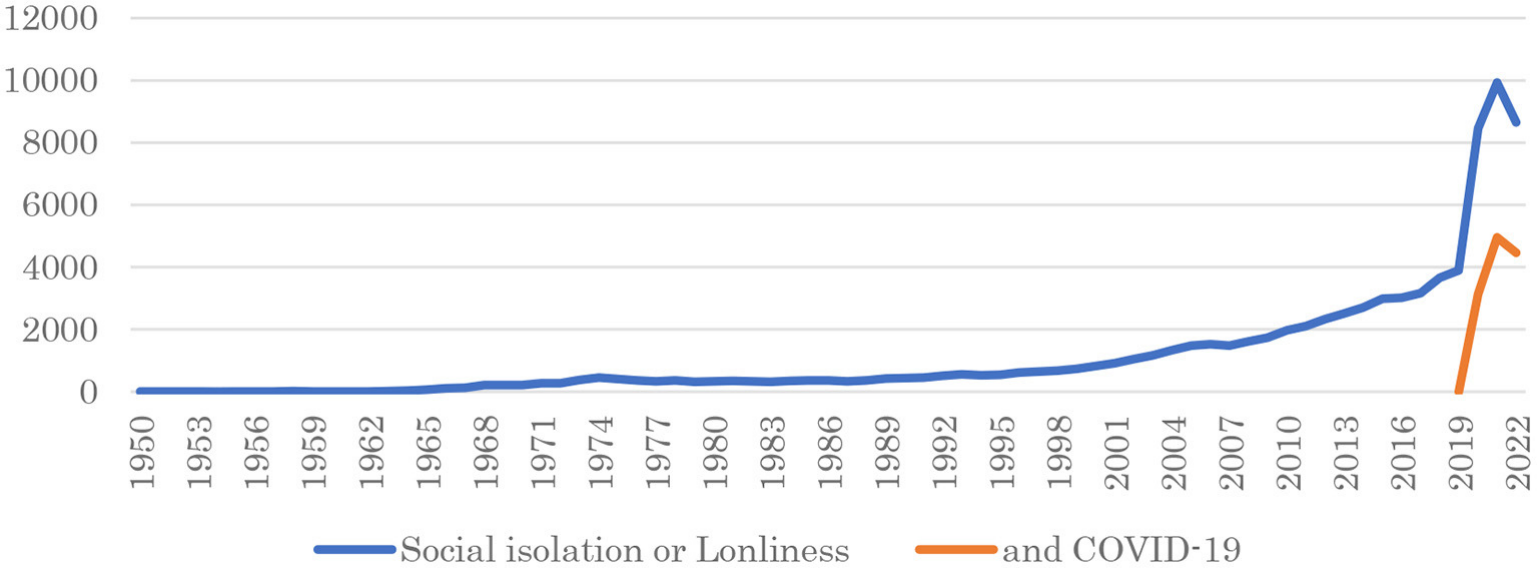
The number of publications about social isolation and loneliness by year. Data were obtained from Scopus (https://www.scopus.com/; accessed on 2022/11/4) through searches for “social isolation” or “loneliness” (blue line) and “social isolation” or “loneliness” plus “COVID-19” (orange line). The figures for 2022 were estimated from the numbers as of 2022/11/4.

This Research Topic was open for submission between 2021/7/16 and 2022/5/31, during the COVID-19 pandemic. Fourteen papers were published on this special topic. Eleven of them had the term “COVID-19” (Bentley et al.; Liu et al.; Okajima et al.; Mansour et al.; Chernova et al.; McDowell et al.; Peng et al.; Lv et al.; Luo et al.; Pilcher et al.) or “coronavirus” (Goddard et al.) in the title, and another study (Lim et al.) tracked changes over time during the pandemic. Two papers were unrelated to the pandemic (Landry et al.; Chiao et al.). There was a report originally submitted to this Research Topic but it was eventually published in another section of this journal (7).

Five studies analyzed young participants aged 13–29 years old. Liu et al. and Okajima et al. studied high school students. Landry et al., Lv et al., and Chiao et al. studied young adults. Even though loneliness is also an important issue for older people (8), none of the studies in this Research Topic examined loneliness in older adult samples. Three studies targeted specific populations: Goddard et al. recruited people with mobility disabilities; Peng et al. studied consumers and their purchasing intentions; and Mansour et al. recruited men for their study. Most papers only focused on loneliness, but four investigated social isolation(Goddard et al.; Landry et al.; Luo et al.; Pilcher et al.).

Most studies analyzed social isolation and loneliness and their impact on mental health symptoms or related issues. Four studies analyzed depression (Liu et al.; Lim et al.; McDowell et al.; Lv et al.), three analyzed distress (Bentley et al.; Liu et al.; Chernova et al.) and anxiety (Okajima et al.; Lim et al.; McDowell et al.), and two analyzed sleep or sleep problems (Okajima et al.; Pilcher et al.). In addition, there were international collaborations during the pandemic (9). Two international studies are reported in this Research Topic (Bentley et al.; Lim et al.). The first study examined the association between loneliness and distress in the early stage of the pandemic in eleven countries (Bentley et al.). A subset of countries (three countries) in the study also repeated the analysis 3 months later and revealed that increased loneliness over time was associated with increased psychological distress (Bentley et al.). The second study examined how social restrictions contributed to the severity of loneliness, depression, and social anxiety in participants recruited from the United Kingdom, Australia, and the United States (Lim et al.). The authors found that as social restrictions eased, loneliness and depression reduced, but there was an increase in social anxiety. Overall, the findings of these studies highlighted how sleep problems, social anxiety, and depression are interrelated (10). However, whether these interrelationships are maintained outside of the context of the pandemic remains unclear without the inclusion of pre-COVID-19 data.

The restrictions imposed on people's lives due to COVID-19 have come as a critical reminder of how fundamental social relationships are to our mental health and wellbeing. Countries around the world observed increasing rates of mental ill health during the pandemic and responded with significant government investment and policy changes to combat it (11).

Overall, the pandemic and its associated consequences for health and wellbeing may have highlighted the critical need for being around people and being meaningfully connected to others around us. A deeper knowledge of loneliness and social isolation is required to allow us to better understand their impact on mental health and wellbeing.

## Author contributions

HK wrote the first draft of the manuscript. ML wrote sections of the manuscript. All authors contributed to the conception, manuscript revision, read, and approved the submitted version.
